# Effect of Serum Albumin Changes on Mortality in Patients with Peritoneal Dialysis: A Joint Modeling Approach and Personalized Dynamic Risk Predictions

**DOI:** 10.1155/2021/6612464

**Published:** 2021-07-21

**Authors:** Merve Basol, Dincer Goksuluk, Murat H. Sipahioglu, Ergun Karaagaoglu

**Affiliations:** ^1^Department of Biostatistics, Abant Izzet Baysal University, Bolu 14030, Turkey; ^2^Department of Biostatistics, Erciyes University, Kayseri 38280, Turkey; ^3^TURCOSA Analytics Solutions Ltd. Co., Erciyes Teknopark 5, 38030 Kayseri, Turkey; ^4^Department of Nephrology, Erciyes University, Kayseri 38280, Turkey; ^5^Department of Biostatistics, Hacettepe University, Ankara 06100, Turkey

## Abstract

Peritoneal dialysis (PD) is a frequently used and growing therapy for end-stage renal diseases (ESRD). Survival analysis of PD patients is an ongoing research topic in the field of nephrology. Several biochemical parameters (e.g., serum albumin, creatinine, and blood urea nitrogen) are measured repeatedly in the follow-up period; however, baseline or averaged values are primarily associated with mortality. Although this strategy is not incorrect, it leads to information loss, resulting in erroneous conclusions and biased estimates. This retrospective study used the trajectory of common renal function indexes in PD patients and mainly investigated the association between serum albumin change and mortality. Furthermore, we considered patient-specific variability in serum albumin change and obtained personalized dynamic risk predictions for selected patients at different follow-up thresholds to investigate the effect of serum albumin trajectories on patient-specific mortality. We included 417 patients from the Erciyes University Nephrology Department whose data were retrospectively collected using medical records. A joint modeling approach for longitudinal and survival data was used to investigate the relationship between serum albumin trajectory and mortality of PD patients. Results showed that averaged serum albumin levels were not associated with mortality. However, serum albumin change was significantly and inversely associated with mortality (HR: 2.43, 95% CI: 1.48 to 4.16). Risk of death was positively associated with peritonitis rate, hemodialysis history, and the total number of comorbid and renal diseases with hazard ratios 1.74, 3.21, and 1.41. There was also significant variability between patients. The personalized risk predictions showed that overall survival estimates were not representative for all patients. Using the patient-specific trajectories provided better survival predictions within the follow-up period as more data become available in serum albumin levels. In conclusion, using the trajectory of risk predictors via an appropriate statistical method provided better predictive accuracy and prevented biased findings. We also showed that personalized risk predictions were much informative than overall estimations in the presence of significant patient variability. Furthermore, personalized estimations may play an essential role in monitoring and managing patients during the follow-up period.

## 1. Introduction

Peritoneal dialysis (PD) is a frequently used and growing therapy for end-stage renal diseases (ESRD). In the 1970s, continuous ambulatory peritoneal dialysis (CAPD) was first discussed by Popovich et al. [1] and became popular through the following decades. With the ongoing clinical developments and advantages (e.g., ease of implementation and cost-effectiveness), PD was accepted as a home-based renal therapy. The worldwide estimated number of PD patients was approximately 150,000 in 2008 [2] and increased to 272,000 in 2017 [3], which suggests that it almost doubled within ten years. It was also reported that PD was approximately 11% of all dialysis modalities in the world [3]. However, the number of PD patients significantly differed among regions, countries, or continents. According to Li et al. [3], peritoneal dialysis use was estimated between 4% and 71.9%, and 5-year survival between 34% and 64% in different countries globally. In Turkey, Sipahioglu et al. [2] estimated 5-year survival rates as 68.8%, and Tekkarismaz and Torun [4] estimated them as 65% in adults, which was higher than other regions reported by Li et al. [3]. Interpreting unadjusted survival rates might be misleading because patients' survival might decrease dramatically with comorbidities and other risk factors (e.g., age at PD initiation, nutritional status, clinical and biochemical outcomes such as hemoglobin level, and white blood cell count). For example, Turkey's five-year survival rates were reported as 36.8% and 79.8% in diabetic and nondiabetic subgroups, respectively, which was found to be significantly different [4].

Predicting survival of a study group was frequently examined using the Kaplan-Meier analysis and/or Cox proportional hazard models. The Kaplan-Meier analysis is practical when researchers aim to compare survival rates between subgroups. The Cox proportional hazard model, on the other hand, allows adjusting estimated survival probabilities by controlling for possible confounders, such as comorbidities, biochemical/chemical and demographic variables, and nutritional status. In practice, PD patients are monitored for nutritional status, kidney functions, and inflammations during the follow-up period. Some variables (e.g., serum bilirubin, albumin and creatinine levels, blood urea nitrogen, and hemoglobin level) are repeatedly measured from PD patients; however, a vast majority of studies ignored repeated measurements for simplicity and used either the Kaplan-Meier or the Cox proportional hazard model based on a single measurement (i.e., baseline or average of multiple records) of related risk factors [3–8]. Although it is possible to examine survival probabilities using averaged values of continuous risk factors associated with PD, the repeated measurements might give better insights for predicting short- or long-term survival probabilities. Association between baseline serum albumin level and mortality is well known [4–9]; however, the relationship between the time-varying serum albumin levels and mortality is a recent and promising research topic. Furthermore, most of the existing studies focused on population-based risk estimations and failed to reflect within-patient variability. In recent years, personalized therapies and risk predictions have become popular in medicine, especially in treating and monitoring chronic diseases [10–12] at the patient level. With recent developments in statistical modeling, it is possible to predict the mortality of PD patients individually by considering patient-specific parameters and changes in common renal function biomarkers (e.g., serum albumin, serum creatinine, and blood urea nitrogen) in time. Furthermore, patient-specific risk predictions for future time points can be updated dynamically whenever new information becomes available.

Serum albumin levels at the beginning of PD initiation or averaged values over the follow-up period are useful to predict survival outcomes in PD patients. However, we believe that the longitudinal change and trend in albumin levels might provide more accurate risk estimations. In this study, we aimed to extend the survival model (generally the Cox proportional hazard model) to repeated measurements (i.e., time-varying predictors) using the joint modeling approach, a novel and recently proposed method [13, 14], to explore the relationship between the longitudinal nature of common renal function biomarkers and survival outcome for patients undergoing PD. In addition, we also aimed to predict personalized dynamic survival estimations via the proposed approach by updating the risk prediction model whenever a new measurement was taken. To the authors' best knowledge, the current study is the first study that examines the joint modeling approach on PD patients in Turkey and is among the few studies published worldwide [15–17]. Furthermore, the current study used personalized risk predictions in PD patients via a joint modeling approach for the first time.

## 2. Materials and Methods

### 2.1. Study Design and Participants

Five hundred eleven patients, who initiated PD at the Erciyes University Nephrology Department, were retrospectively included in the study [2]. Patients were followed starting from PD initiation to death, kidney transplant, termination of PD, lost to follow-up due to withdrawal or unknown reasons, or end of the study, whichever occurred first. Participants were initiated PD between the years 1995 and 2007; however, the follow-up period continued until 2016. Among 511 patients, 423 patients were included in the study according to exclusion criteria such as (i) patients that recovered kidney functions and needed no further treatment, (ii) aged below 18 years at PD initiation, (iii) survived less than 90 days, and (iv) patients that had missing data for most of the variables of interest. In addition, we excluded three patients who were transferred to PD from kidney transplantation and three patients who have no values in the repeatedly measured response variable (e.g., serum albumin levels and blood urea nitrogen). Finally, 417 patients were included in the study (see Supplementary Figure 1 for a complete study flowchart).

### 2.2. Clinical Outcome

The primary clinical outcome was mortality due to PD. In this retrospective cohort study, patients' data, including the demographic (e.g., age at PD initiation, body mass index, gender, the existence of comorbid diseases, dialysis history, and PD modality) and clinical/biochemical measurements (e.g., serum bilirubin, albumin, hemoglobin levels, white blood cell count, calcium, triglyceride, high- and low-density lipoprotein levels, glomerular filtration rate, and parathyroid hormone), were collected from medical records. Repeated measurements of nutritional, biochemical, and clinical outcomes were recorded within the follow-up period. The association between the magnitude of change in trajectories of longitudinal biomarkers and mortality was investigated. Patients who died during the PD process or three months after transferring to hemodialysis (HD) were considered PD-related deaths. Sipahioglu et al. [2] found that several biochemical parameters significantly changed between groups defined as low and high according to the transport property of the peritoneal membrane. Furthermore, 5- and 10-year survival estimates were lower in high transport characteristics. Hence, we used a similar grouping strategy as in Sipahioglu et al. [2] and examined survival outcomes by adjusting transport characteristics of the membrane.

The effect of comorbid and renal diseases on mortality is well established among PD patients via the Charlson comorbidity index (CCI) [18, 19]. Our study could not calculate the CCI because the data for some comorbid diseases, essential for the CCI calculation, were either unmeasured or missing. Hence, we preferred to use the total number of comorbid and renal diseases observed in a patient as an independent predictor to reflect the influence of comorbidity on mortality. Cardiovascular disease, lung disease, hepatitis, diabetes mellitus, glomerulonephritis, hypertension, and polycystic kidney disease were considered to calculate the total number of comorbid and renal diseases; therefore, it takes values between 0 and 7.

### 2.3. PD Modalities

Prior renal replacement therapy (RRT) of patients was either hemodialysis or kidney transplantation. At PD initiation, participants were assigned to one of the PD modalities, CAPD with a twin-bagged system or automated PD (APD). The prescription of CAPD was 4x 2 L exchanges as long as no sign of inadequate dialysis was observed. Dialysate fluids containing (i) 1.36%, 2.27%, or 3.86% glucose; (ii) amino acids; or (iii) icodextrin were used according to the clinical needs of patients. The catheter exit site was regularly dressed with polyvinylpyrrolidone iodine (Poly-Iodine) to avoid catheter exit site and tunnel inflammations and peritonitis. Many of the patients and their caregivers were informed about the sterilization techniques at baseline PD initiation and during PD. However, we could not collect information from medical records whether patients and their caregivers, who were enrolled before the year 2000, were trained about sterilization techniques or not.

### 2.4. Statistical Analysis

Statistical analyses were performed on the R language environment (version 4.0.2, URL: https://cran.r-project.org). Numerical variables were summarized using the mean and standard deviations and median and quartiles (or minimum and maximum) for normally and nonnormally distributed variables, respectively. Categorical variables were summarized with frequencies and percentages. Normality of data was assessed using the graphical (histogram, Q-Q plots, etc.) and analytical (Shapiro-Wilk's normality test) approaches.

The relationship between the trajectory of longitudinal biomarkers and mortality was evaluated using a joint model, which is comprised of two submodels: (i) a linear mixed effect (LME) model for evaluating the longitudinal biomarker and (ii) a Cox proportional hazard model for mortality. The joint modeling approach is aimed at predicting the effect of the longitudinal biomarkers on mortality while adjusting both the longitudinal and survival outcomes for possible confounders. At first, a univariate Cox proportional hazard model and an LME model were used to find a list of significant predictors from among biochemical, clinical, nutritional, and demographic variables. Next, the survival and longitudinal submodels were combined using a joint model, and model parameters were estimated simultaneously. The analyses were conducted in R using the JMbayes [13] package developed explicitly for joint modeling of longitudinal and survival processes.

In the joint modeling, the longitudinal nature of the response variable is modeled using an LME model given in

(1) $$\begin{array}{c} y_{it}=m_{it}+\epsilon_{i}=\beta^{T}X+b^{T}Z+\epsilon_{i}, \end{array}$$

where *X* and *Z* are the vectors of fixed and random effects with the vectors of regression parameters *β* and *b*, respectively, and *ϵ*_*i*_ is the random error term of the *i*th patient. The fitted curves of trajectories from the longitudinal model were included as a time-varying covariate in the survival part through an association parameter. The survival submodel can be specified as in

(2) $$\begin{array}{c} h_{i}\left(t\right)=h_{0}\left(t\right)\exp\left(\gamma_{i}^{T}\;w_{i}+\alpha m_{it}\right), \end{array}$$

where *w*_*i*_ is the vector of baseline covariates of the *i*th patient associated with mortality, *γ*_*i*_^*T*^ is the vector of model parameters, and *m*_*it*_ is the fitted trajectory of serum albumin levels calculated via an LME model for the *i*th patient at time *t* [14]. Here, *α* is the association parameter between longitudinal and survival submodels. The effect of the longitudinal biomarkers (e.g., serum albumin levels and BUN) is incorporated into the survival model after adjustment for possible confounders through an LME model. If the parameter *α* is statistically insignificant, then there is no significant association between mortality and the longitudinal biomarker.

PD-related deaths might be associated with several biomarkers such as serum albumin, serum creatinine, and blood urea nitrogen. This paper mainly focused on serum albumin and used albumin levels at 6-month intervals as a longitudinal response. Joint modeling results of blood urea nitrogen and serum creatinine were given as supplementary. We separated two patients, one censored and the other dead, as an independent test set for evaluating the predictive performance of the built model. These patients were randomly selected from participants with similar baseline serum albumin levels but trajectories in opposite directions, i.e., better nutritional conditions for the censored and worse nutritional conditions for the dead in time. Finally, patient-specific dynamic survival predictions were obtained for the patients in the test set at different time points to forward. The level of statistical significance was set at *p*  < 0.05 in all analyses.

## 3. Results

The current study included 417 patients who underwent PD, of which 364 (87.3%) were treated with CAPD. Prior RRTs were hemodialysis in 54 (12.9%) patients and kidney transplantation in 3 (0.7%) patients. Many of the participants were first-ever PD patients (86.4%). The mean age of patients at PD initiation was 45.92 ± 14.33 years. The most frequent causes of end-stage renal disease were DM (34.8%), hypertension (14.9%), and glomerulonephritis (9.1%) (Table 1). The median follow-up duration of patients was 30.05 (range: 3 to 137) months. Within the follow-up period, 86 (20.6%) patients died primarily due to cardiovascular events (21.8%), peritonitis, and other infections (26.4%). The most common comorbid disease was CVD (22.1%), followed by hepatitis (14.4%) and lung disease (3.1%).

Table 2 shows the results of univariate and multivariate Cox proportional hazard models. We used the univariate Cox model results to define a list of independent predictors of mortality. Repeatedly measured variables were averaged over the follow-up period and included in the Cox models. According to the multivariate Cox proportional hazard model results, averaged albumin levels, BUN, WBC, transport characteristic of the peritoneal membrane, and age at PD initiation were not associated with mortality when adjusted for the remaining risk factors. However, the final risk estimates and conclusions were carried out using the survival part of the joint model (Table 3), and the results were compared with the multivariate Cox model.

**Figure 1:**
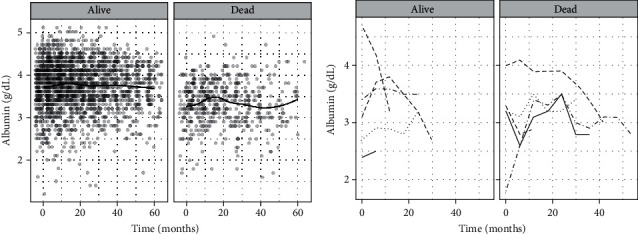
Trajectory of serum albumin levels: (a) all patients and (b) randomly selected 5 patients in each group.

The change in serum albumin levels in time is given in Figure 1. Serum albumin levels were higher in alive patients and slightly changed over time. However, in dead patients, inclines and declines in serum albumin levels were observed (Figure 1(a)). Furthermore, there was significant variability in serum albumin trajectories between patients.  Figure 1(b) shows the patient-specific changes in albumin levels for five randomly selected patients within a 5-year follow-up period. The multivariate Cox proportional hazard model (Table 2) failed to consider time-varying nutritional status and kidney functions of patients because averaged values were used. In the joint modeling (Table 3), the patient-specific trajectory of albumin levels was fitted to an LME model, and adjusted serum albumin levels were associated with mortality through the survival submodel (see Supplementary Figure 2 for the complete list of adjusted variables in each submodel).

According to the joint modeling results, the effects of BUN, age at PD initiation, the total number of comorbid diseases, peritonitis rate, and WBC on time-varying serum albumin were found statistically insignificant. The estimated serum albumin levels were 0.27 mg/dL lower in high transporters. It increased 0.028 mg/dL and 0.279 mg/dL with every 1 mg/dL increase in serum creatinine and serum calcium levels, respectively. There was a significant and inverse relationship between mortality and the trajectory of serum albumin levels. A 1 g/dL decrease in the adjusted albumin levels at a time point *t* (i.e., a unit change in the adjusted trajectory) resulted in 2.43 times higher risk of death (95% CI: 1.48 to 4.16). A patient's risk was 3.21 times (95% CI: 1.94 to 5.70) higher if initiated PD from HD and increased 1.24 (95% CI: 1.10 to 1.40) times with every 1 mg/dL decrease in the serum creatinine levels. Finally, the risk of death increased 1.41 times with every increase in the total number of comorbid and renal diseases, 1.07 times with a 1 kg/m^2^ increase in BMI, and 1.74 times with a 1-unit increase in peritonitis.

### 3.1. Dynamic Prediction of Patient-Specific Survivals

**Figure 2:**
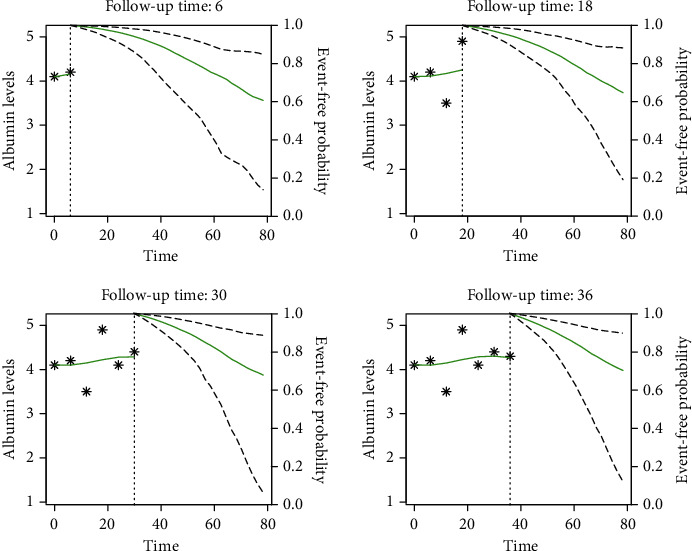
Dynamic survival predictions: patient survived 97+ months (age: 55, gender: female).

**Figure 3:**
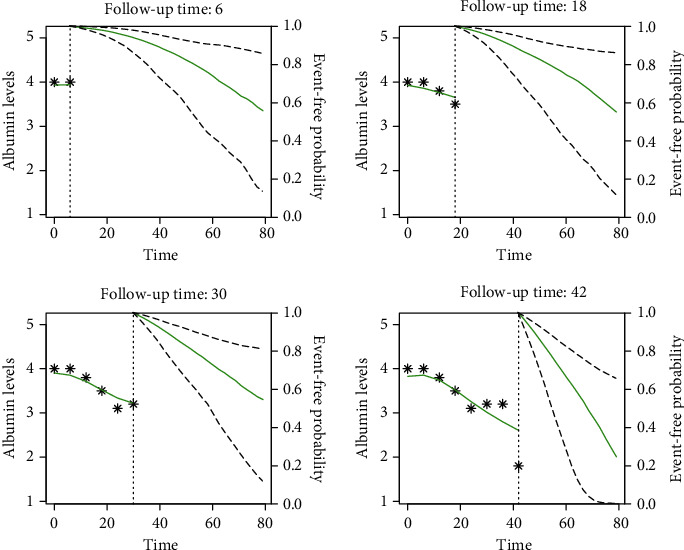
Dynamic survival predictions: patient died at 80 months (age: 63, gender: female).

The Cox proportional hazard model gives population-based risk estimates and survival predictions. However, the survival outcomes may differ between patients depending on the patient-specific nutritional status and kidney functions during the follow-up period (Figures 1(b), 2, and 3). Therefore, the personalized and dynamic risk predictions, which are estimated at different time thresholds within the follow-up period, is preferred. Figures 2 and 3 show the dynamic survival predictions of selected test samples. These figures are generated at different follow-up thresholds of a patient to investigate the effect of serum albumin trajectories on mortality. Serum albumin levels were presented on the left-hand side of the dashed vertical line, and given that the patient survived at the selected follow-up time point, the predicted survival probability of that patient for the future time points up to 80 months presented on the right-hand side of the vertical axis of each graphic.

Serum albumin levels were higher in the survived patient while it got worse in time for the dead patient. In the early times of follow-up, predicted survival probabilities at 80 months were approximately 60% for both patients. However, better survival predictions were obtained as more information about serum albumin trajectory was collected from patients, which was 70% for the survived and 20% for the dead (Figures 2(d) and 3(d)).

## 4. Discussion

This study evaluated the association between serum albumin trajectories and mortality in PD patients using the joint modeling approach. Our findings showed that the changes (increases and decreases) in serum albumin over time were strongly and significantly associated with mortality after adjustment for the risk factors including serum creatinine, serum calcium, white blood cell count, age at PD initiation, peritonitis rate, BMI, prior RRT, the total number of comorbid and renal diseases, and transport characteristic of the peritoneal membrane (Supplementary Figure 2). The joint modeling approach provided more accurate survival estimates in PD patients as compared to the Cox proportional hazard model. The reasons why the joint modeling approach was more accurate may be as follows: (i) it used the cumulative and historical information of serum albumin, (ii) the true and unobserved trajectory of serum albumin was estimated by the linear mixed model and adjusted before associating with mortality, (iii) model parameters were jointly calculated by considering the association between longitudinal and survival processes, and (iv) the trajectory of serum albumin levels was estimated at the patient level using the patient-specific estimating equations, i.e., random effects. The risk of death increased by 2.43 times (95% CI: 1.48 to 4.16) with every 1 g/dL decrease in the adjusted serum albumin at any time point. Therefore, an efficient dietary program controlling the protein and energy intake could improve the nutritional status of PD patients, keep the serum albumin at a steady state, slow renal disease progression, and decrease the risk of death.

Serum albumin is an indicator of the protein status and calorie intake, amount of peritoneal and renal albumin loss, adequacy of dialysis, and systemic diseases. Low serum albumin at baseline PD, one time point after PD initiation, or averaged over time, was associated with mortality and technical failure in the previous studies [6, 20–23]. However, few studies, including the current study, have focused on the trajectory of albumin levels [15, 17, 24]. Flanigan et al. [25] reported serum albumin values unchanged over the follow-up period. Khoshhali et al. [24] found that serum albumin was monotonically decreasing over time within three years of the follow-up period. However, this trajectory was reported for the group total. In the current study, we observed different patterns in the overall trajectory of serum albumin levels for dead and censored populations. Serum albumin was almost constant during the follow-up period in alive patients. In dead patients, on the contrary, it increased after PD initiation for the following two years, reached steady-state for a short-term, and decreased until 48 months (Figure 1). We also observed an increase in albumin levels after 48 months, which might be due to better nutritional facts. However, we did not have sufficient data to associate it with nutritional status because there were only 34 patients after 40 months in the dead group. This increase might be a side effect of the small sample size on the fitted curve of serum albumin trajectory. Therefore, it should be justified on a larger study group that included more patients who survived more than 40 months.

Serum albumin represents the nutritional status of PD patients, and it is correlated with other nutritional indices in a connection system. Serum creatinine, which is proportional to skeletal muscle and dietary protein intake, is an indicator of nutritional status in the maintenance and monitoring of PD patients [2, 26]. In our study, serum creatinine was positively associated with serum albumin. Serum creatinine levels might increase with the increasing amount of skeletal muscle due to better nutritional status, and serum albumin may be positively affected by the amount of serum creatinine. Therefore, serum creatinine was used for two different purposes: (i) to adjust serum albumin and (ii) to predict mortality. The risk of death increased 1.24 times for every 1 mg/dL decrease in serum creatinine.

Higher transport status was associated with an increased risk of death [2, 27, 28]. The high transportation group generally has excess peritoneal loss of albumin which may lead to worse nutritional status and increase the risk of death. Greater loss of protein into dialysate and inhibition of appetite due to increased absorption of glucose might be the reason for malnutrition. According to the results of a meta-analysis study [27], transport status increased the risk of death between 21.9% and 77.3% depending on the severity of transport status. In our study, serum albumin levels were estimated at 0.27 g/dL lower in higher transporters, resulting in a 27.2% higher risk of death.

Peritonitis is the major complication of PD and closely related to technical failure [16, 21, 27] and all-cause mortality [29–32]. Several studies associated low serum albumin at baseline PD initiation [33, 34] and change in follow-up period [35, 36] with increased risk of peritonitis. Decreases in serum albumin before PD initiation or during PD may cause malnutrition and poor health conditions in the long term and increase the risk of peritonitis. In this study, the mean peritonitis rate was 0.58 (median: 0.32) and associated with subsequent risk of death, a 74% higher risk for every 1-episode/patient-year increase in the peritonitis rate.

Other risk factors associated with mortality were prior RRT, BMI, and the total number of renal and comorbid diseases. BMI was associated with mortality, a 7% higher risk of death with every 1 kg/m^2^ increase in BMI at baseline PD initiation. This study included three patients transferred to PD from kidney transplantation. We excluded these patients due to the very small sample size and evaluated the effect of hemodialysis history (no vs. yes) on mortality. The risk of death increased by 3.21 times (95% CI: 1.94 to 5.70) if a patient was initiated HD before PD, which was similar to the findings of previous studies [37–39]. The majority of these patients had a vascular access problem. Therefore, the increased risk of death was possibly influenced by hypercoagulability or atherosclerosis in these patients.

This study evaluated the effect of comorbidity using the total number of concurrent comorbid and renal diseases observed in a patient. In practice, each comorbid or renal disease may be independently associated with mortality. However, using the total number of diseases may be more informative since the risk of death may increase with the existence of multiple diseases. Comorbidity was positively associated with mortality, increasing the risk of death 41% with one extrarenal or comorbid disease observed in a patient. Patients died mostly due to cardiovascular disease (CVD). The reason why CVD accounted for the majority of death might be because the protective effect of serum albumin significantly reduced with decreasing serum albumin, which increases the risk of CVD [40].

The nutritional status of patients may be influenced by several factors, including social deprivation, medication history, and dietary factors. Therefore, serum albumin trajectories are expected to be significantly different between patients (see Figure 1(b)), which indicated a significant patient variability. The built joint model returned the model parameters in two partitions: the main effects and the random effects, which can be used to evaluate population-based and patient-specific risk estimations, respectively. We selected two patients, one dead and one censored, to evaluate the predictive performance of personalized risk estimations. Both patients were monitored within the follow-up period, and the personalized survival estimates up to 80 months were predicted dynamically at different time thresholds as new measurements became available. Even though the serum albumin at baseline PD initiation was similar in both patients, we observed better nutritional status in censored and worse in dead patients within the follow-up period. Also, serum albumin instantly decreased for the dead patient at 12 months. The predicted survivals were approximately 60% in both patients when the baseline serum albumin was considered. However, we estimated 20% survival for the dead and 80% for the censored at 80 months when the serum albumin trajectory was considered (Figure 3(c)). Dynamic predictions clearly showed that the predicted survivals are highly affected by the longitudinal trajectory of serum albumin and other patient characteristics. This finding has shown the importance of using personalized risk estimations in the care management and monitoring of PD patients. In nephrology, personalized risk estimations obtained at different time points may help physicians taking patient-specific decisions such as changing the dietary factors, drug usage, and change of modality. Furthermore, based on the built joint model, a web- or computer-based and real-time running decision support system can be developed to support physicians in their decisions. Such a system could be used to monitor patients, obtain dynamic and personalized risk estimations instantly at any time during the follow-up period, and generate patient-specific reports.

This study mainly focused on the trajectory of serum albumin and evaluated the association with mortality. However, it is possible to build joint models using other renal function indexes. We provided the joint modeling results of serum creatinine and blood urea nitrogen as a supplementary. Although we associated serum creatinine and blood urea nitrogen with mortality in the Cox proportional hazard model, the joint modeling results showed that the trajectory of blood urea nitrogen and serum creatinine was not associated with mortality (Supplementary Tables 1 and 2,1). Hence, it is not required to build a joint model for blood urea nitrogen or serum creatinine.

### 4.1. Limitations, Possible Sources of Bias, and Generalization Issues

This study has some limitations. It is a single-centered retrospective cohort study, and the results may include center-specific effects. There are unmeasured parameters, which are possibly associated with serum albumin and mortality, including residual kidney function, serum potassium, malnutrition-inflammation complex syndrome (MICS), comorbidity (the Charlson comorbidity index, CCI), dialysis adequacy, race, and kidney transplantation as prior RRT. We did not have enough data to adjust the abovementioned confounders, specifically for the older medical records. Therefore, the validity of our findings should be confirmed in the presence of unmeasured confounders.

An important part of data was collected based on practice before the recommendations that were released by the International Society for Peritoneal Dialysis (ISPD) in 2017. Our study may partially meet the criteria for avoiding peritonitis and other inflammations. The peritonitis rate in our study group was similar to the literature. Nonetheless, it may be possible to decrease the peritonitis rate and other inflammations by considering the current care management and recommendations. Therefore, the association between peritonitis and mortality should be validated on a recent dataset.

Another limitation and bias arose from associating the comorbidity with mortality. We were unable to calculate CCI due to insufficient data. We tried to reflect the influence of comorbidity by using the total number of renal and comorbid diseases. Although our approach associated comorbidity with mortality, the results may not be as accurate as CCI. We used a primitive predictor of comorbidity; therefore, our findings should be validated using the CCI, whenever it can be calculated.

This study has the potential to be improved. Multivariate extension of the joint modeling may improve the model performances. The multidimensional trajectory of longitudinal biomarkers (e.g., serum albumin, BUN, and serum creatinine) may be adjusted for confounding factors, and the adjusted trajectory of multivariate responses may be associated with mortality [9]. This paper studied the simpler model, and the multivariate joint modeling was not covered because it requires a larger sample size. We leave this as another research topic to be applied to a larger study group.

## 5. Conclusions

The Cox proportional hazard model and Kaplan-Meier method are easy to implement and practical to present population-based risk predictions. However, these methods poorly explain the effect of change in the time-varying response variable (e.g., serum albumin levels) on mortality. Also, such methods are not capable of considering patient variability. We implemented an advanced modeling strategy, called the joint model, to evaluate the association between time-varying covariates and mortality as well as considering the patient variability. According to the findings, we suggest using the joint model, a more advanced alternative to the Cox proportional hazard model, for accurately associating the time-varying biomarkers with mortality.

This study indicated that the trajectory of serum albumin was a more potent predictor than baseline or averaged serum albumin. Furthermore, the serum albumin trajectory was the only significant predictor of mortality from others, including the serum creatinine and blood urea nitrogen trajectories. In practice, the independent risk predictors that we considered in this study were collected regularly from PD patients. Therefore, the proposed modeling strategy can be easily applied to other PD patients.

In summary, the current study and the modeling approach used have several advantages: (i) it incorporates the change in albumin levels in time into Cox proportional hazard model; hence, it uses all the information in the modeling process; (ii) it allows to predict patient-specific mortality along with a population-based estimation; and (iii) finally, it allows the monitoring of patients using dynamic survival predictions for future time points. This study, as being among the first studies, contributes to the peritoneal dialysis literature by approaching the survival analysis from a different perspective and making use of personalized predictions of mortality for the first time.

## Figures and Tables

**Table 1:** Biochemical, clinical, and demographic findings of the study group (*n* = 417).

Characteristic	Summary statistics^∗^
Age	45.92 ± 14.33
BMI at PD initiation (kg/m^2^)	23.63 ± 4.11
Gender, male	238 (57.1)
PD modality, CAPD	364 (87.3)
Prior RRT	
First-ever PD	363 (87.1)
Hemodialysis (HD)	54 (12.9)
Cause of ESRD^†^	
Diabetes mellitus (DM)	145 (34.8)
Glomerulonephritis	38 (9.1)
Hypertension	62 (14.9)
Polycystic kidney disease (PKD)	19 (4.6)
Unknown	112 (27.1)
Other	41 (9.8)
Comorbidity	
Cardiovascular disease	92 (22.1)
Lung disease	13 (3.1)
Hepatitis	60 (14.4)
Total number of comorbid/renal diseases^a^	1 [0-5]
Peritonitis rate (episodes/patient-year)	0.32 [0, 5.33]
Serum albumin (g/dL)^††^	3.57 [1.75, 4.75]
Blood urea nitrogen (mg/dL)^††^	53.6 [18.5, 119]
Serum creatinine (mg/dL)^††^	7.35 [2.2, 18.48]
Serum calcium (mg/dL)	9.12 ± 0.71
WBC (×1000/mm^3^)^††^	7.44 [3.6, 14.22]
Parathyroid hormone (pg/mL)	89 [2.0, 2059.8]
GFR (mL/min/1.73 m^2^)	7.99 [0, 27.8]

^∗^Summarized using mean ± standard deviation, frequency (percentage), or median [minimum, maximum] where appropriate. BMI: body mass index; WBC: white blood cell counts; ESRD: early-stage renal disease; GFR: glomerular filtration rate. ^a^Total number of comorbid and renal diseases observed in a patient. ^†^Patients might have more than one disease causing ESRD. ^††^Averaged over follow-up period.

**Table 2:** Univariate and multivariate Cox proportional hazard models.

Parameters	Univariate model		Multivariate model^∗∗^	
	HR (95% CI)	*p* value	HR (95% CI)	*p* value
Serum albumin (g/dL)^†^	0.35 (0.21, 0.59)	<0.001	0.59 (0.31, 1.13)	0.116
Blood urea nitrogen (mg/dL)^†^	0.98 (0.96, 1.01)	0.068	0.99 (0.98, 1.02)	0.978
Serum creatinine (mg/dL)^†^	0.77 (0.71, 0.85)	<0.001	0.82 (0.73, 0.92)	<0.001
Serum calcium (mg/dL)^†^	0.93 (0.67, 1.30)	0.665	—	—
Parathyroid hormone, log-scaled (pg/mL)^†^	0.88 (0.72, 1.09)	0.253	—	—
WBC (×1000/mm^3^)^†^	1.17 (1.06, 1.30)	0.003	1.05 (0.93, 1.18)	0.444
Glomerular filtration rate, log-scaled (GFR)	0.94 (0.86, 1.03)	0.159	—	—
Transferred from HD (yes)	1.84 (1.10, 3.10)	0.021	3.07 (1.71, 5.50)	<0.001
No. of diseases^††^	1.79 (1.43, 2.23)	<0.001	1.34 (1.03, 1.17)	0.031
Age at PD initiation	1.04 (1.02, 1.06)	<0.001	1.02 (0.99, 1.04)	0.125
BMI at PD initiation (kg/m^2^)	1.09 (1.04, 1.15)	<0.001	1.09 (1.02, 1.16)	0.009
Peritonitis rate	2.26 (1.65, 3.09)	<0.001	1.83 (1.30, 2.59)	<0.001
Transport characteristic (high)	1.56 (1.01, 2.40)	0.045	0.95 (0.59, 1.54)	0.841

^†^Averaged values over follow-up period were used. ^††^A total number of comorbid and renal diseases observed in a patient. ^∗∗^Variables with *p* values < 0.10 in the univariate model were included in the multivariate model.

**(a):** 

Longitudinal part (linear mixed effects)^∗^		
Variable	Estimate (95% CI)	*p* value
Serum creatinine^a^	0.028 (0.012, 0.042)	0.004
Serum calcium^a^	0.279 (0.198, 0.358)	<0.001
BUN^a^	-0.0001 (-0.004, 0.004)	0.968
Age at PD initiation	-0.005 (-0.009, 0.001)	0.081
Number of diseases^b^	0.0006 (-0.063, 0.057)	0.998
Peritonitis rate	-0.051 (-0.123, 0.018)	0.163
WBC	-0.029 (-0.058, 0.014)	0.103
Transportation characteristic (high)^†^	-0.270 (-0.414, -0.207)	<0.001

**(b):** 

Survival part (Cox proportional hazard)^∗∗^			
Variable	Estimate (95% CI)	HR (95% CI)	*p* value
Serum creatinine^a^	-0.212 (-0.336, -0.096)	1.24 (1.10, 1.40)^†††^	<0.001
BUN^a^	0.004 (-0.016, 0.025)	1.01 (0.98, 1.03)	0.668
Age at PD initiation	0.016 (-0.001, 0.34)	1.02 (0.99, 1.40)	0.069
WBC	0.061 (-0.073, 0.183)	1.06 (0.93, 1.20)	0.323
HD history (yes)^†^	1.165 (0.665, 1.740)	3.21 (1.94, 5.70)	<0.001
Number of diseases^b^	0.345 (0.09, 0.661)	1.41 (1.09, 1.94)	0.009
BMI	0.071 (0.011, 0.148)	1.07 (1.01, 1.16)	0.010
Peritonitis rate	0.556 (0.140, 0.891)	1.74 (1.15, 2.44)	<0.001
Transportation characteristic (high)^†^	-0.095 (-0.467, 0.276)	0.91 (0.63, 1.32)	0.622
Albumin (*α*)^††^	-0.889 (-1.425, -0.392)	2.43 (1.48, 4.16)^†††^	<0.001

HR: hazard ratio; BMI: body mass index (kg/m^2^); GFR: glomerular filtration rate. ^†^Model parameters were obtained for the group given in parenthesis. ^††^Albumin levels are estimated from longitudinal part of joint model. ^†††^Hazard ratios were estimated for 1-unit decrease in corresponding predictors. ^a^Averaged over the follow-up period. ^b^The total number of comorbid and renal diseases observed in a patient.

## Data Availability

The peritoneal dialysis data used to support the findings of this study are available from the corresponding author upon request.

## Supplementary Materials

Supplementary 1 Supplementary Table 1: joint modeling results—longitudinal response is serum creatinine levels (*n* = 415). Supplementary Table 2: joint modeling results—longitudinal response is blood urea nitrogen levels (*n* = 415).

Supplementary 2 Supplementary Figure 1: detailed study flowchart. Supplementary Figure 2: variable selection results of the joint models.
