# Supplementary material for: Effect of Serum Albumin Changes on Mortality in Patients with Peritoneal Dialysis: A Joint Modeling Approach and Personalized Dynamic Risk Predictions
Source: Biomed Res Int. 2021 Jul 21;2021:6612464. doi: 10.1155/2021/6612464 (PMC8319732; doi:10.1155/2021/6612464)
Supplement: Supplementary 1 — Supplementary Table 1: joint modeling results—longitudinal response is serum creatinine levels (n = 415). Supplementary Table 2: joint modeling results—longitudinal response is blood urea nitrogen levels (n = 415). [file 6612464.f1.docx]

| **Supplementary Table 1**. Joint modeling results – longitudinal response is serum creatinine levels (n = 415) | | | | | | |
| --- | --- | --- | --- | --- | --- | --- |
| **Longitudinal part (Linear mixed effects)*** | | | | | | |
| Variable | | Estimate (95% CI) | |  | p value | |
| Serum albumin ^a^ | | 0.597 (0.211, 1.005) | |  | 0.002 | |
| Serum calcium ^a^ | | 0.463 (0.088, 0.829) | |  | 0.017 | |
| BUN ^a^ | | 0.049 (0.034, 0.065) | |  | < 0.001 | |
| HD history (Yes) ^†^ | | 0.179 (-0.565, 0.905) | |  | 0.630 | |
| BMI | | 0.067 (0.011, 0.126) | |  | 0.013 | |
| log(PTH) | | 0.126 (-0.076, 0.332) | |  | 0.221 | |
| log(GFR) | | -0.397 (-0.503, -0.287) | |  | < 0.001 | |
| Number of diseases ^b^ | | -0.293 (-0.536, -0.053) | |  | 0.018 | |
| Transportation characteristic (High) ^†^ | | -0.478 (-0.896, -0.058) | |  | 0.019 | |
| Age at PD initiation | | -0.065 (-0.082, -0.049) | |  | < 0.001 | |
| **Survival part (Cox proportional hazard)**** | | | | | | |
| Variable | Estimate (95% CI) | | HR (95% CI) | | | p value |
| HD history (Yes) ^†^ | 1.219 (0.634, 1.735) | | 3.38 (1.88, 5.67) | | | < 0.001 |
| Age at PD initiation | 0.027 (0.003, 0.046) | | 1.027 (1.003, 1.05) | | | 0.011 |
| Number of diseases ^b^ | 0.457 (0.219, 0.685) | | 1.58 (1.24, 1.98) | | | < 0.001 |
| BMI | 0.076 (0.012, 0.144) | | 1.08 (1.01, 1.15) | | | 0.029 |
| BUN ^a^ | 0.001 (-0.026, 0.023) | | 1.001 (0.97, 1.02) | | | 0.970 |
| Serum albumin ^a^ | -0.348 (-0.867, 0.416) | | 1.42 (0.66, 2.38) ^†††^ | | | 0.340 |
| WBC | 0.022 (-0.083, 0.136) | | 1.02 (0.92, 1.14) | | | 0.718 |
| Peritonitis rate | 0.669 (0.376, 1.009) | | 1.95 (1.46, 2.74) | | | < 0.001 |
| Transportation characteristic (High) ^†^ | 0.1175 (-0.327, 0.434) | | 1.12 (0.72, 1.54) | | | 0.449 |
| HD history (Yes) ^†^ | 1.186 (0.678, 1.809) | | 3.28 (1.97, 6.10) | | | < 0.001 |
| Creatinine ($\boldsymbol{\alpha}$) ^††^ | -0.071 (-0.174, 0.021) | | 1.07 (0.98, 1.19) ^†††^ | | | **0.136** |
| **HR**: Hazard ratio; **BUN**: Blood urea nitrogen (mg/dL); **BMI**: Body mass index (kg/m^2^);  **WBC**: White blood cell counts; **PTH**: Parathyroid hormone; **GFR**: Glomerular filtration rate  ^†^ Model parameters were obtained for the group given in parenthesis.  ^††^ Serum creatinine levels are estimated from longitudinal part of the joint model.  ^†††^ Hazard ratios were estimated for 1-unit decrease in corresponding predictors.  ^a^ Averaged over follow-up period.  ^b^ Total number of comorbid and renal diseases observed in a patient. | | | | | | |

| **Supplementary Table 2**. Joint modeling results – longitudinal response is blood urea nitrogen levels (n = 415) | | | | | | | |
| --- | --- | --- | --- | --- | --- | --- | --- |
| **Longitudinal part (Linear mixed effects)*** | | | | | | | |
| Variable | | Estimate (95% CI) | |  | p value | |  |
| Serum creatinine ^a^ | | 1.631 (1.602, 1.662) | |  | < 0.001 | |  |
| Serum albumin ^a^ | | 0.176 (0.009, 0.330) | |  | 0.047 | |  |
| Age at PD initiation | | 0.007 (-0.013, 0.001) | |  | 0.071 | |  |
| Number of diseases ^b^ | | -0.615 (-0.693, -0.532) | |  | < 0.001 | |  |
| Transportation characteristic (High) ^†^ | | -1.22 (-1.362, -1.071) | |  | < 0.001 | |  |
| log(GFR) | | -0.331 (-0.364, -0.297) | |  | < 0.001 | |  |
| BMI | | 0.763 (0.745, 0.782) | |  | < 0.001 | |  |
| **Survival part (Cox proportional hazard)**** | | | | | | | |
| Variable | Estimate (95% CI) | | HR (95% CI) | | | p value | |
| Serum creatinine ^a^ | -0.180 (-0.280, -0.084) | | 1.20 (1.09, 1.32) ^†††^ | | | < 0.001 | |
| Serum albumin ^a^ | -0.651 (-1.631, -0.098) | | 1.92 (1.10, 5.11) ^†††^ | | | 0.040 | |
| Age at PD initiation | 0.016 (-0.002, 0.033) | | 1.02 (0.99, 1.03) | | | 0.090 | |
| HD history (Yes) ^†^ | 1.026 (0.526, 1.480) | | 1.69 (2.79, 4.39) | | | < 0.001 | |
| Number of diseases ^b^ | 0.179 (-0.037, 0.405) | | 1.19 (0.96, 1.50) | | | 0.103 | |
| BMI | 0.088 (0.038, 0.146) | | 1.09 (1.04, 1.16) | | | < 0.001 | |
| WBC | 0.062 (-0.051, 0.202) | | 1.06 (0.95, 1.22) | | | 0.304 | |
| Peritonitis rate | 0.548 (0.254, 0.798) | | 1.73 (1.29, 2.22) | | | < 0.001 | |
| Transportation characteristic (High) ^†^ | -0.086 (-0.625, 0.481) | | 0.92 (0.54, 1.62) | | | 0.723 | |
| BUN ($\boldsymbol{\alpha}$) ^††^ | -0.015 (-0.035, 0.006) | | 1.02 (0.99, 1.04) | | | **0.168** | |
| **HR**: Hazard ratio; **BUN**: Blood urea nitrogen (mg/dL); **BMI**: Body mass index (kg/m^2^);  **WBC**: White blood cell counts; **GFR**: Glomerular filtration rate  ^†^ Model parameters were obtained for the group given in parenthesis.  ^††^ Blood urea nitrogen levels are estimated from longitudinal part of joint model.  ^†††^ Hazard ratios were estimated for 1-unit decrease in corresponding predictors.  ^a^ Averaged over follow-up period.  ^b^ Total number of comorbid and renal diseases observed in a patient. | | | | | | | |
