# Supplementary material for: Effect of Serum Albumin Changes on Mortality in Patients with Peritoneal Dialysis: A Joint Modeling Approach and Personalized Dynamic Risk Predictions
Source: Biomed Res Int. 2021 Jul 21;2021:6612464. doi: 10.1155/2021/6612464 (PMC8319732; doi:10.1155/2021/6612464)
Supplement: Supplementary 2 — Supplementary Figure 1: detailed study flowchart. Supplementary Figure 2: variable selection results of the joint models. [file 6612464.f2.docx]

| 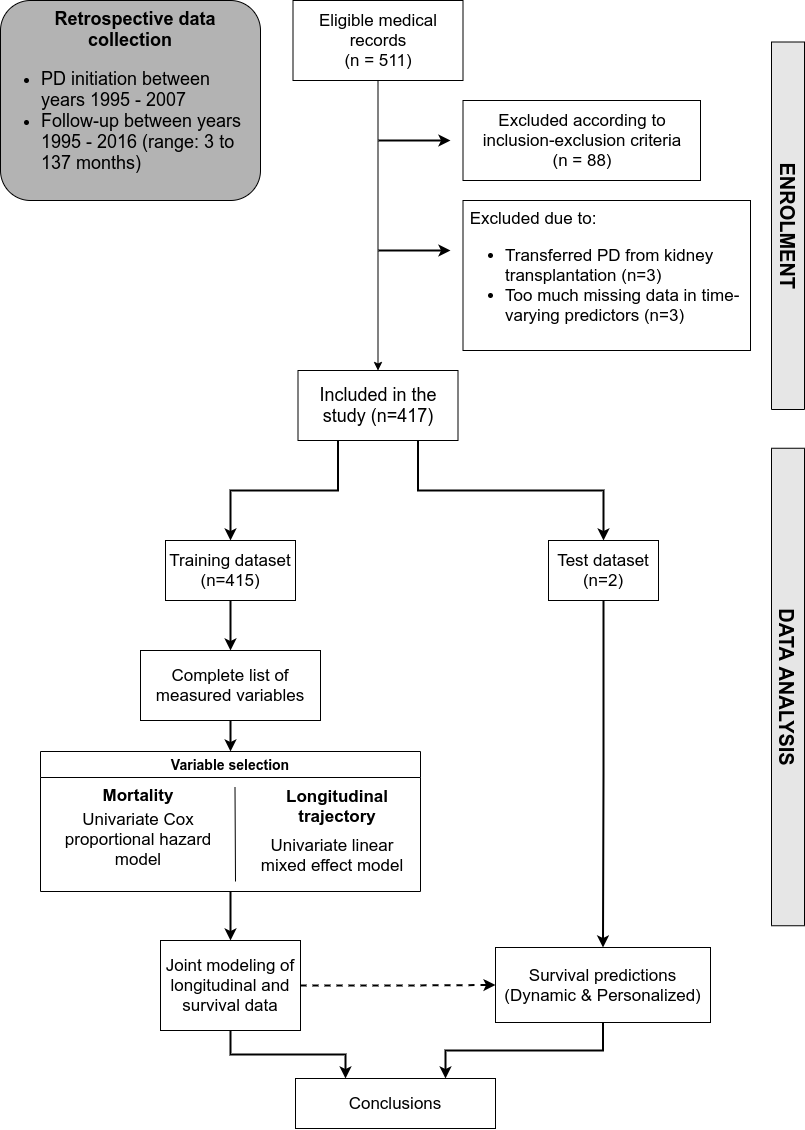 |
| --- |
| **Supplementary Figure 1.** Detailed study flowchart. |

| 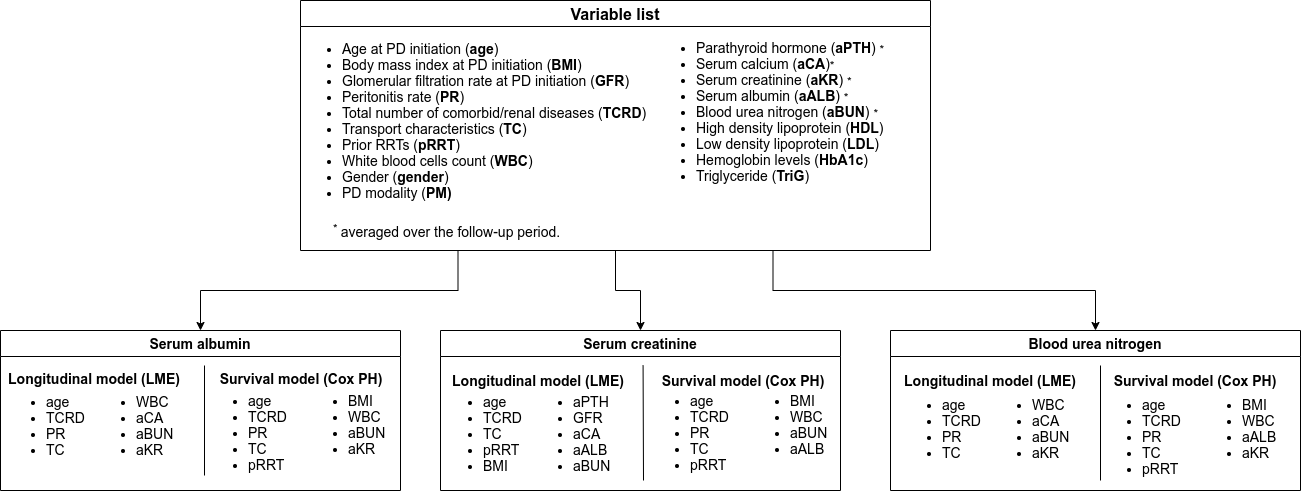 |
| --- |
| **Supplementary Figure 2.** Variable selection results of the joint models |
